# Acknowledgment of Reviewers

**DOI:** 10.2188/jea.JE20220314

**Published:** 2022-12-05

The following scientists assisted the journal by reviewing manuscripts during the period November 1, 2021 to October 31, 2022.

We gratefully acknowledge their critical evaluation and valuable assistance in selecting articles for publication.

**Table:** 

Abe, Sarah K.
Abe, Takumi
Amagasa, Shiho
Ando, Emiko
Ando, Yuichi
Anzai, Tastuhiko
Arima, Yuzo
Asakura, Keiko
Baba, Sachiko
Cable, Noriko
Chen, Jie
Esaki, Yuichi
Fujii, Ryosuke
Fujiyoshi, Akira
Fukui, Keisuke
Fukushima, Noritoshi
Gando, Yuko
Gianfagna, Francesco
Goto, Aya
Goto, Tadahiro
Hagiwara, Yasuhiro
Hamada, Shota
Hanazato, Masamichi
Harada, Kazuhiro
Hayashi, Katsuma
Hedman, Linnea
Hirata, Aya
Hori, Megumi
Horikoshi, Yuho
Hosozawa, Mariko
Ichikawa, Shuhei
Ide, Kazushige
Iizuka, Ai
Ikeda, Ai
Ikeda, Nayu
Imaeda, Nahomi
Imamura, Haruhiko
Inoue, Kosuke
Ishikawa-takata, Kazuko
Ishimaru, Miho
Iwagami, Masao
Iwasaki, Masanori
Iwasaki, Motoki
Kabasawa, Keiko
Kachi, Yuko
Kajiwara Saito, Mari
Kakizaki, Masako
Kamada, Masamitsu
Kanamori, Satoru
Kanda, Hideyuki
Kase, Tetsuo
Kashino, Ikuko
Katanoda, Kota
Katsuura-Kamano, Sakurako
Kawado, Miyuki
Kihara, Tomomi
Kikuchi, Hiroyuki
Kikuya, Masahiro
Kim, Jeongseon
Kim, Kyeezu
Kim, Mi Kyung
Kino, Shiho
Kishimoto, Hiro
Kitabatake, Yoshinori
Kobayashi-Cuya, Kimi
Kogure, Mana
Kojima, Masayo
Kojima, Taro
Kumamaru, Hiraku
Kurita, Noriaki
Kutsuna, Satoshi
Li, Yuanying
Lin, Yingsong
Lv, Xiao
Makita, Noriko
Matsumoto, Naomi
Matsuyama, Yusuke
Mezawa, Hidetoshi
Mi, Minami
Mitsui, Takahiko
Mitsunami, Makiko
Mitsutake, Seigo
Miyagawa, Naoko
Miyake, Yoshihiro
Miyawaki, Atsushi
Mizoue, Tetsuya
Momma, Haruki
Morimoto, Akiko
Morisaki, Naho
Morita, Ayako
Morita, Ichizo
Murakami, Keiko
Nakagomi, Atsushi
Nakajima, Mikio
Nakamura, Mieko
Nakano, Shiori
Nakata, Yoshio
Nakaya, Naoki
Nakaya, Tomoki
Nemoto, Yuta
Nexø, Mette
Nishioka, Norihiro
Noda, Tetsuya
Noma, Hisashi
Oba, Shino
Obayashi, Kenji
Ochi, Manami
Ohfuji, Satoko
Ohnishi, Hirofumi
Okada, Yohei
Okami, Yukiko
Okamoto, Etsuji
Okubo, Ryo
Okumura, Yasuyuki
Orihara, Shunichiro
Orui, Masatsugu
Osaki, Yoneatsu
Osawa, Itsuki
Ozasa, Kotaro
Park, Boyoung
Park, Hyesook
Rasmussen, Svein
Renson, Audrey
Rossi, Marta
Rudolph, Jacqueline
Saika, Kumiko
Sakamaki, Kentaro
Sakurai, Masaru
Sanada, Takahiro
Sato, Koryu
Sato, Shinichiro
Sato, Shuntaro
Schlaud, M
Seino, Satoshi
Shi, Ju-Fang
Shiba, Sakurako
Shimizu, Sayuri
Shimizu, Yuji
Shinozaki, Tomohiro
Smith, Louisa
Sobue, Tomotaka
Sugimoto, Kumi
Suma, Shino
Suzuki, Harumitsu
Suzuki, Kohta
Suzuki, Yuka
Tabuchi, Takahiro
Tachimori, Hisateru
Taguri, Masataka
Takagi, Daisuke
Takahashi, Kunihiko
Takaku, Reo
Takeuchi, Jiro
Takeuchi, Masato
Takeuchi, Yoshinori
Takimoto, Hidemi
Tanaka, Hirokazu
Tanaka, Junko
Tanaka, Tomoki
Tang, Julian
Taniyama, Yukari
Taoka, Kazuki
Togawa, Kayo
Tomata, Yasutake
Tsuji, Taishi
Tsukinoki, Rumi
Tsuno, Kanami
Ueda, Yutaka
Uemura, Hirokazu
Usui, Takeshi
Utsunomiya, Takeshi
Watanabe, Daiki
Weng, Lu-Chen
Wu, Na
Xia, Changfa
Yamada, Michiko
Yamagishi, Kazumasa
Yamakita, Mitsuya
Yatsuya, Hiroshi
Yazawa, Aki
Yokomichi, Hiroshi
Yokoyama, Yuri
Yoneoka, Daisuke
Yorifuji, Takashi
Yoshida, Lay-Myint
Yoshioka, Eiji
Yumoto, Tetsuya
Zaidi, Jaffer
Zhang, Tao

